# Introductory of Prof. Marsh

**Published:** 1856-12

**Authors:** 


### DEPARTMENT OF SELECTIONS.

### INTRODUCTORY OF PROF. MARSH.

The Professor selected for his theme "Medical Conservatism," a subject of deep moment and great importance to the profession. In the discussion of this subject in a truthful and candid manner, there is little to be gleaned calculated to awaken zeal in the mind of the student just upon the threshold of a profession fraught with toil, sacrifices and discomforts. These last, of themselves, are sufficient to discourage the beginner without shaddowing his pathway with doubts and darkness in relation to the truths of the science whose depths he is about to fathom. With those who believe that introductories should rather prove encouraging than otherwise, that they should arouse ardor and awaken zeal in an undertaking, at best requiring moral courage to enter upon; with such we say, the selection of the topic on the occasion would be regarded as not the most advisable. But for our part, we believe, we never lose anything in avowing truth at all times, and under all circumstances—taking the con-consequences of such avowal. If, upon a truthful statement of facts in advance, any one is deterred from a prosecution of the science of medicine, now is the time for his retirement and retreat, both for himself and the profession.

The following we select from the Address, which we regard as well timed:

"Previous, however, to entering upon the discussion of the topic thus selected, I cannot forbear, gentlemen, to ask your attention to the consideration of the position you now occupy as students of medicine. I do not doubt that the most, perhaps all of you, have canvassed the matter thoroughly and well, and are fully satisfied that, in the medical profession, you are to find your legitimate vocation. And yet, believing as I do, that in the decision of this question, you are performing an act second to none other in life, in importance,—I ask you again, even at this late hour, to give it candid and considerate examination and again make up your decision.

"Be careful that you rightly appreciate and fully comprehend the matter upon which you are to decide. Call up all and everything incident to both the study and the practice of the profession you contemplate selecting, as the one to which you are to devote yourselves, and placing that which is of a pleasant, by the side of that which is of an unpleasant nature, then settle in your own minds if your appropriate place be here or elsewhere.

"Examine yourselves to know if your tastes and ambitions will here be satisfied, and your intellectual appetites here find food suited to their wants. Ask yourselves if medical science is that mistress to whom you are willing to devote your affections, and for whom you are ready, nay, anxious to count labor, and anxieties, and sacrifices, even to the hazard of life itself as nothing, if thereby you may but vindicate her title to pre-eminence in beauty, virtue and truth. Is your mind so constituted as to be able to estimate the advancement of scientific truth, your own growth in knowledge, and the elevation and improvement of the healing art, as of higher worth, not to others, but to yourselves, than all other personal or selfish interests? Remember, that to a very small minority of medical men are the pecuniary rewards of their profession superior, or even equal to those which result from equal labor in any one of the other professions and trades. Other rewards are ours, but are you determined, and that too, from intelligent consideration, that these are of a character to gratify your desires?

"All these and many more questions of a like character are to be debated by you, and affirmatively passed upon before you may, with safety to yourselves, and to the honor and credit of the profession, enter upon the path you have chosen. Upon your decision the happiness and the usefulness of a lifetime, and that your own, will rest. And more, upon that decision will depend, to a certain extent, the character of the medical profession, for, in my opinion, legitimate medicine has more to fear from the admission into its ranks of unworthy members, than from anything which enemies from without can do."

In the following, the learned Professor very plainly intimates the folly of attempting to write down the "one idea systems" of the day, and maintains that they are on the increase. Now, while we admit that there is a greater variety of *isms*, and some of them without precedent in absurdity, yet it is questionable whether, comparatively speaking, there is a greater sum of real quackery than heretofore; and there is another question which cannot be very well determined, we admit, and that is, whether these systems would not have multiplied, acquired much more power, and increased in their prevalence, had not the journals kept a running and effective fire upon them. Legitimate medicine has been defended and its power preserved and increased; it has been made aware of the kind and character of the foe in whatever deceptive garb it has appeared, and its strength and influence has kept it from acquiring ascendancy. Such results are the legitimate fruits of the promulgation of truth. But to the extract:

"It is common to hear expressions of surprise and alarm at the rapid increase of the numerous "one idea systems" of the day, and the journals are full of ingenious projects for lessening the evil, and whole books have been written for the same object. And to what purpose? The monster not only will not "down at our bidding," but still grows on, gathering day by day, new strength and larger influence. And wherefore? Is it matter of astonishment that we cannot write or talk down what we are all the time directly, although unintentionally, laboring to build up? Do but listen to the chief boast of each and all of these irregular systems—that the majority of their practitioners are renegades from the regular profession! I shame to say it, but yet so far as diplomas and licenses go, they have the right so to boast. What wonder then, that in view of this fact, those who have the real good of the medical profession at heart, should begin in alarm—almost in despair—to ask "who shall guard the *shepherds*?""

There is so much of plain truth in the following, that we can not forbear making the extract, and trust that the suggestion will be received by medical men who admit students into their offices for private instruction:

"The evil is not yet, however, beyond the reach of remedies. But to cure, here, is possible only by observing the indication, "removal of cause." Let the profession generally, and particularly those to whom it is allotted to act as teachers, set firmly their faces against the indiscriminate encouragement of those seeking too thoughtlessly to enter the profession. Hold out no inducements to engage in the study of medicine, to any save those whose abilities and ambition are of a character to make them valuable acquisitions to its numbers, and hearty laborers in the cause of true medical progress.

"It is full time that we should have learned, what every day's experience has for long years been teaching, that intellectual weakness and indolence are incompatible with success in medical pursuits. To one who thinks at all, it cannot but be a manifest impossibility, that the study and practice of such a science as ours shall be prosecuted with benefit to themselves, the profession or society, by those who have no more love of intellectual pursuits, than to be satisfied merely with a parchment permission to dispense drugs and collect fees, nor hardly enough of ambition and industry to enable them to secure even this. If eternal vigilance be the price of liberty, it is equally the price of eminent professional worth, and it is but a sequence flowing legitimately out from the cause, when we see skepticism as to the competency of medical science springing from that ignorance of its principles and precepts which is the result of indolence or incapacity. And, moreover, to these sluggards, the many exclusive systems of the day hold out inducements which they have neither the ability, nor yet a desire to forego, and they *fall* out of their nominal position in the ranks of regular medicine, to become, not so much active in the promulgation of their new fath, as bitter and venomous villifiers of that which they never comprehended. These are the examples to which non-professional men point as testimony of the worthlessness of medical science, and of the incapacity of medical men."

The following passage commends itself to our judgment, as much because of the truths it contains, as the eloquent manner in which it is expressed. How common it is for some minds to abandon an old truth for some new coined opinion, gilded over by the blinding glitter of sophistry. Such are always changing their store of knowledge instead of carefully selecting and acquiring new material, by a close process of examination in which the worthless is rejected, and truth approved to be stored away:

"To one class of minds every truth is valuable, because it is truth, and, moreover, because it adds one more link to that great chain, which, as they conceive, binds and makes of all truth, whether of philosophy, science or religion, one great whole, beautiful, harmonious, sublime. To them, every step of genuine progess is of inestimable worth, since it is an advance, a nearer approximation towards the solution of the great problem, "what is truth?" All knowledge and wisdom, and all goodness, are to them attractive, not because they are of so much value in the market of other men's esteem, but because of their influence upon themselves, in lifting them upwards and farthering them onwards towards that perfection of all, for which the soul is ever reaching and ever struggling to attain. Nor yet are they restlessly anxious of progressing as to choose to retrograde rather than stand still where present advance seems impossible. In their ardor for that which is new, they are not wont to forget the value of what they already possess. Caution marks their steps, and they are not easily deceived into mistaking the shadow for the substance, nor into forsaking the tried lessons of experience, for the loose vagaries of imagination. Obstacles in their way are expected to yield only to patient and judiciously expended labor. Faith follows only upon due examination and critical investigation of each proposition claiming belief."

We commend the following to the consideration of the reader as eminently worthy of considerate attention, with this single remark, that, although observation and experience may prove fallacious by poor, erring mortal man, yet withal, they are the basis of science, without which we could not make deductions approximating to truth. A series of observations is but a tissue of experience; for the same phenomenon observed again and again, is but continued experience from time to time in scuh phenomena.

"But while in all departments of knowledge, the influences of which I have spoken, have had their effect in giving existence to, and sustaining the various parties, sects and schools of politics, religion, philosophy, and science—medicine, is now, and has been ever, the most obnoxious to them.

"It is true the medical profession has always been ranked as one of the learned professions, and has, moreover, deserved such rank. And yet it has never been impossible to find within it and almost equally prominent, both learning and ignorance, philosophy and superstition, science and charlatanry.

"The same classes of men enter here, and exert more or less of influence in giving tone and character to its institutes and doctrines as in other of the departments of life. Medicine has its rational thinkers, its dreamers, its blind worshippers of the past, and its nobodies.

"In other branches of science, there is a possibility of successful generalization. A few general principles underlie the whole; and all phenomena may be traced back to these for their explanation. In the ascertaining of these principles, it is true, great obstacles supervene, and but very few are able to successfully prosecute investigation and inquiry, to the accomplishing of this end. It requires a high order of intellect to be able to rise, from fact to the principle whence it derives its origin. A falling apple was a key to the secret of gravitation to the mind of a Newton only. Nor for the purposes of science are the majority of men qualified by mental habits, properly to observe even the most common and simple facts, nor to detail the results of personal experience. Hence the tardy growth of the natural sciences.

"But in addition to all these difficulties, medical knowledge has been embarrassed by those unavoidable circumstances which surround every problem involving the phenomena of life, and set at defiance all ordinary attempts to appreciate their influence.

"Successful generalization being impossible to any large extent, we are driven to dependence upon the lessons of experience. Gradually winning its way from empiricism, towards a rational system, exposed to the influence of fluctuating opinions, and peculiarly obnoxious, to be effected by theories and hypotheses of all kinds, it is hardly strange that its progress should have been tardy and irregular, and that, to-day, we cannot claim for it anything like that demonstrative and scientific certainty which are the attributes of other sciences.

"Observation and experience are ever liable to be fallacious. As has been very justly remarked, "the human understanding is not a mere faculty of apprehension, but is affected more or less by the will and the passions; what man wishes to be true, that he too easily believes to be so," and physic has, of all sciences, the least pretentsions to proclaim itself independent of the empire of the passions.

"But freely acknowledging this, it is nevertheless, true, that enlightened experience, when rightly construed, may, and does furnish results, every whit as valuable, and in every sense as reliable as those following accurate scientific deductions. And to him who reads philosophically, medical history, there is no fact more forcibly impressed upon the mind than that scientific researches of modern times, have oftimes but resulted in the confirmation of the truths "digged from out the hard mine of experience," by the ancient fathers. Those very truths, had, perhaps, long been derided or unappreciated, had exercised no influence in the founding of medical systems and theories."

After defining "Conservatism," and then administering some excellent advice, the Professor gives a few very interesting *recipes* used in times long since gone bye, and then furnishes us with a few of more modern origin and in use by a class of most learned and profound medical philosophers of the present progressive era. There is one prevalent idea which would be reduced to fact, and that is, that doctors take their own medicine, provided such pharmacopœia were adopted into general use.

"Conservatism is defined the preservation of that which is established, but it does not demand that we should yield submission to that which, although established, will not bear investigation. To do this, were to make the satire of LeSage too true, that "death has two wings: on one are painted war, plagues, famine, fire, shipwreck, with all the other miseries that every instant offer him new prey. On the other, you behold a crowd of young physicians, about to take their degree before him. Death proceeds to dub them doctors, having first made them swear, never in any way to alter the established practice of physic."

"We are to remember that *facts* are changeless, but *opinions* variable—the first (if reliably ascertained and honestly related,) are always authoritative; the latter may or may not be so. Remember that the object of the true physician is, not to learn what is the established practice, but to learn what is the established truth.

"And in regard to new systems which offer themselves for your approval, recollect that they are to be rejected or adopted not because they conflict or agree with prevailing systems, but because they fail or succeed when tried by the tests.

"Reject nothing simply because it conflicts with previously entertained opinions, nor yet adopt anything because it promises much. "Prove all things: hold fast that which is good," is an injunction no less applicable to medical theories, than to morals and religion. A true and legitimate conservatism, is a genuine eclecticism. Do not think that because interested partizans claim perfection for what they advocate, that the absurdity of the claim necessarily argues a total want of truth in them or their theories. "There is a soul of good" oftimes "in things evil," and it is a fact worthy of note, that many of the most valuable truths of modern science are indebted for their discovery to the efforts of those engaged in the pursuit of the most insane conceits and hypothesis.

"Chemistry were to-day, perhaps, only in is infancy, but for the hypothetical philosopher's stone, which, Erastus says, was "a mere imposture, a *non ens*, digg'd out of that broody hill belike this goodly golden stone is," *ubi nascitur ridiculus mus.* And the Materia Medica is indebted for many of its most valuable articles to the efforts put forth for the discovery of the fabled elixir of life.

"So too, of some of the exclusive systems of the present day, they have, in some things, proved beneficial to legitimate medicine, because in the zeal to establish a single principle or uphold a peculiar dogma, they have stumbled upon, now and then, a valuable truth, or proven the incorrectness of some previously holden opinion.

"But it would be no less an error to adopt the whole of a *new*

theory, simply because it possesses an element of truth in its composition, than to go back to those long since exploded, because of the same reason.

"In our day, we have other tests than those possessed by our fathers, by which to try the truth or falsehood of that appealing to us for belief. Before chemistry had revealed the secret of the peculiar composition of the human body, and thrown its light upon the mysterious changes transpiring in the system, in health and disease, and taught the true relations of remedies to peculiar diseased states, it is hardly wonderful that such absurd conceits as the following should have received the assent not only of the foolish, but of the learned and of philosophers.

"The "Bezoar is a preventative from pestilential air, and a remedy for the small pox and other contagious diseases; it is also reckoned proper against vertigos, epilepsics, palpitation of the heart, jaundice, colic, gravel, dysentery, and against poisons."

"Of tea, Bontekoe says, "it fortifies the memory and all the intellectual faculties—against fever there is no better remedy than forty or fifty cups of tea swallowed immediately one after another; the slime of the pancreas is thus washed off."

"Or another remedy, very much esteemed, viz: "a ring made out of the hoof of an ass's *right fore foot*," or "the emanation from a spider" confined "in a nut shell, lapped in silk and carried about the person to prevent ague."

"But in the light of to-day, you will say we shall hardly be called upon to exercise our judgments upon such silly propositions. It would indeed hardly seem possible, and yet mankind are nothing changed from what they were, and precisely in proportion as they cast away the aids which science offers, do they at once fall backward into like fallacies and follies!

"Thus, in *Jahrs Nouvelle Pharmacopœ Homœpathique*, published in Paris, in 1841, in the list of Materia Medica, we find the most disgusting remedies recommended, among which, I mention only a few, as follows:

"*Lachesis*, or poison of rattlesnakes, *formaoca rufa*, or red ants, *aradea diadema*, a species of spider, *rana bufo*, the toad, the *ecrivisees*, or fresh water crab, which is to be *pounded alive* in a mortar, and reduced to a fine paste, and then taken as directed.

"Toads, lizzards, cockchafers, and other reptiles, are brayed (alive,) &c., &c. Could ancient credulity go farther, or lead to more disgusting results. And thus, it will ever prove, when men loose sight of the rational fact, that it is dangerous to reject the lights of science and experience. For, as has been truly said, "there is a maturity of the human mind acquired from generation to generation in the *mass*, as there is in the individual man," and to cast aside the instrumentalities by which such maturity has been secured, is but to return to childhood and puling infancy."

The address will commend itself to the reader for its originality, the vigor and terseness with which the thoughts are expressed, its boldness and independence. The author has shown himself not only in possession of great mental vigor but of habits of close thought and observation.

Prof. Marsh is not one of those who believe that knowledge comes by intuition, but is a man of research, and is constantly reaching forward for new truths. Such ardent minds, not satisfied with what they already know, are sure to attain to eminence in their profession, and this is the sure destiny of the author of the address from which we have taken a few extracts.—Ed.

